# ‘Great in theory’: Women’s care experiences in relation to Australia’s national maternity Strategy—Qualitative survey responses

**DOI:** 10.1371/journal.pone.0319249

**Published:** 2025-04-15

**Authors:** Paula Medway, Alison M. Hutchinson, Linda Sweet

**Affiliations:** 1 School of Nursing and Midwifery & Centre for Quality and Safety Research, Institute for Health Transformation, Faculty of Health, Deakin University, Victoria, Australia; 2 Department for Health and Wellbeing, Government of South Australia, Australia; 3 Barwon Health, Geelong, Victoria, Australia; 4 Western Health, St Albans, Victoria, Australia; University of Gothenburg: Goteborgs Universitet, SWEDEN

## Abstract

**Background:**

The provision of woman-centred maternity care in Australia is guided by a national Strategy released in November 2019 titled *Woman-centred care: Strategic directions for Australian maternity services* (the Strategy). The Strategy upholds four values (safety, respect, choice, and access) that underpin twelve principles of woman-centred care.

**Aim:**

To examine the maternity care experiences of women in Australia and explore how these align with the stated values and principles of the Strategy.

**Methods:**

A national online survey was undertaken between February and June 2023. Women who received all their maternity care in Australia since 1 January 2020 were invited to participate. The survey consisted of predominantly closed questions; however, six open-text questions were included to give participants the opportunity to provide in-depth responses about the Strategy and its values. This paper presents a qualitative content analysis of the free-text responses.

**Findings:**

A completed survey was submitted by 1750 eligible participants, of whom 1667 provided 3562 qualitative responses included in this analysis. These showed that while the definition of safety provided in the Strategy favours physically safe care, the survey participants preferred a definition that was more holistic, providing for emotional and psychological safety. Participants expressed the need for respectful relationships with their maternity care providers where they felt listened to and heard. They wanted to be made aware of their choices and to have their maternity care decisions supported without coercion. Participants also desired access to continuity of care, particularly with midwives, and greater access to mental health support across the maternity care episode.

**Conclusion:**

The intent of the national Strategy has not yet been fully realised. A nationally coordinated response is required if the Strategy is to move from policy to practice, ensuring that women in Australia receive true woman-centred maternity care as intended.

## Introduction

Each year in Australia, around 300,000 women give birth [1]. On a number of internationally accepted safety and quality measures, Australia is considered a safe country in which to give birth [2]. Maternity care in Australia is delivered through a mixture of public and private services in the form of eleven main models of care as defined by the validated [3] Maternity Care Classification System [4] (See Table 1). The four most common models of care are public hospital maternity care, private obstetrician specialist care, general practitioner obstetric shared care, and midwifery group practice (MGP) caseload care [4].

**Table 1:** Models of Australian maternity care as described by the Australian Institute of Health and Welfare’s Maternity Care Classification System [4].

Model of care	Description
Private obstetrician specialist care	Antenatal care is provided by a private specialist obstetrician. Intrapartum care is provided in either a private or public hospital by the private specialist obstetrician in collaboration with hospital midwives. Postnatal care is usually provided in the hospital by the private specialist obstetrician and hospital midwives and care by midwives may continue in the home, hotel or hostel. Most models in this category provide continuity of carer across the whole maternity period.
Private midwifery care	Antenatal, intrapartum and postnatal care is provided by a privately practicing midwife or group of midwives in collaboration with doctors in the event of identified risk factors. Antenatal, intrapartum and postnatal care could be provided in a range of locations including the home. This category is used when the designated maternity carer is a privately practicing midwife but is not used if the model of care is shared care between a private midwife and a hospital as part of a formal arrangement.
General Practitioner obstetrician (GPO) care	Antenatal care is provided by a GPO. Intrapartum care is provided in either a private or public hospital by the GPO in collaboration with the hospital midwives. Postnatal care is usually provided in the hospital by the GPO and hospital midwives.
Shared care	Antenatal care is provided by a community maternity service provider (doctor and/or midwife) in collaboration with hospital medical and/or midwifery staff under an established agreement. Can occur both in the community and in hospital outpatient clinics. Usually includes an agreed schedule of antenatal care between the two providers. Intrapartum and early postnatal care usually takes place in the hospital, by hospital midwives and doctors, often in conjunction with the community doctor or midwife (particularly in rural settings).
Combined care	Antenatal care is provided by a private maternity service provider (doctor and/or midwife) in the community. Intrapartum and early postnatal care is provided in a public hospital, by hospital midwives and doctors. Postnatal care may continue in the home or community by hospital midwives. Usually exists without a shared care agreement, so there is no agreed schedule of visits between providers and the private provider does not provide any care in hospital.
Public hospital maternity care	Antenatal care is provided in hospital outpatient clinics (either onsite or outreach) by midwives and/or doctors. Care may also be provided by a multidisciplinary team. This is the broadest model category and includes a range of models of care from those led by midwives that target low risk women to those led by obstetricians that target women with obstetric risk factors such as diabetes. IP and postnatal care is provided in hospital by midwives and doctors in collaboration. Postnatal care may continue in the home or community by hospital midwives.
Public hospital high risk maternity care	Antenatal care is provided to women with medical high risk/complex pregnancies by public hospital maternity care providers (specialist obstetricians and/or maternal-fetal medicine subspecialists in collaboration with midwives). Intrapartum and postnatal care is provided by hospital doctors and midwives. Postnatal care may continue in the home or community by hospital midwives. This category is *not* used for obstetric-led clinics (models of care) such as those designed for women with diabetes or with risk factors such as high BMI. Models requiring obstetric input but not multi-disciplinary specialised care are classified as *public hospital maternity care*.
Team midwifery care	Antenatal, intrapartum and postnatal care is provided by a small team of rostered midwives in collaboration with doctors in the event of identified risk factors. Intrapartum care is usually provided in the hospital or birth centre. Postnatal care may continue in the home or community by the team midwives.
Midwifery group practice caseload care	Antenatal, intrapartum and postnatal care is provided within a publicly funded caseload model by a known primary midwife with secondary backup midwives providing cover and assistance, in collaboration with doctors in the event of identified risk factors. Antenatal care and postnatal care is usually provided in the hospital, community or home with intrapartum care in a hospital, birth centre or home. This category provides continuity of carer across the whole maternity period.
Remote area maternity care	Antenatal and postnatal care is provided in remote communities by a remote area midwife (or nurse) or group of midwives, sometimes in collaboration with a remote area nurse and/or doctor. Antenatal care may also be provided via telehealth or fly-in-fly-out clinicians in an outreach setting. Intrapartum and early postnatal care is provided in a regional or metropolitan hospital (often involving temporary relocation prior to labour) by hospital midwives and doctors.
Private obstetrician and privately practising midwife joint care	Antenatal, intrapartum and postnatal care is provided by a privately practising obstetrician and midwife from the same collaborative private practice. Intrapartum care is usually provided in either a private or public hospital by the privately practising midwife and/or private obstetrician in collaboration with hospital midwifery staff. Postnatal care is provided in hospital and may continue in the home.

For five years, *Woman-centred care: Strategic directions for Australian maternity services* (the Strategy) has been the overarching document informing the provision of safe, woman-centred maternity care in Australia. The Strategy was released by the Council of Australian Governments (COAG) Health Council in November 2019 and provides guidance to states and territories to ‘support Australia’s high-quality maternity care system and enable improvements in line with contemporary practice, evidence and international developments’ [2 p.4]. Unlike previous maternity care policies in Australia, the Strategy is not time-limited and seeks to ensure that women receive maternity care that is safe, equitable, and respectful of each woman’s individual needs and preferences [2]. The Strategy was developed following two rounds of public nationwide in-person consultation with over 600 health professionals, consumers, and maternity care providers; while over 900 organisations and individuals provided written submissions [2].

Woman-centred maternity care is the key intention and sits at the centre of the Strategy; this is supported by four values of safety, respect, choice, and access [2]. These values underpin twelve principles for woman-centred care provision, which are aligned with the White Ribbon Alliance’s Respectful Maternity Care Charter [2,5]*.* In addition to the values and principles are 13 strategic directions that guide the states and territories in how the Strategy should be operationalised. The Strategy is comparable to national maternity policies and plans found in similar English-speaking countries [6]. In a 2023 scoping review of the literature, the four values in the Strategy were found to align with those of women receiving maternity care in Australia [7]. Since its 2019 release, however, very little has been published to indicate how effective the Strategy has been in guiding the provision of safe, respectful woman-centred maternity care nationally. A baseline report released in 2022 suggested that most key stakeholders (which included clinicians, policy-makers and consumers) held the view that its implementation had not progressed well, partially due to the impacts of the COVID-19 pandemic [8].

A healthy start to life is a known social determinant of health [9] and can impact how an individual experiences health across the lifespan [10], so the importance of high-quality maternity care cannot be overstated. The Strategy has the capacity to improve maternity care provision in Australia if implemented as intended, especially amongst the named priority populations - Aboriginal and Torres Strait Islander women, women from rural and remote areas, and women from culturally and linguistically diverse backgrounds. With rising rates of birth trauma and intervention in Australian maternity services [8,11], it is timely to examine women’s experiences of care as compared to the Strategy. Therefore, the aim of this research was to explore women’s perceptions of their maternity care according to the values and principles of the Strategy, by examining the qualitative responses of women who participated in a national online survey [12].

## Methods

### Study design

For this study we used a naturalistic inquiry approach to present the qualitative data from a cross-sectional, national online survey [12]. Naturalistic inquiry is a systematic process to explore phenomena as close to their natural state as possible. It is based on the assumption that meaning and context are inseparable, but this enables the researcher to understand the context in which the meaning has been generated [13]. A web-based survey design is of benefit when participants are geographically dispersed [14], as occurs in Australia. As this study required a national approach and aimed to capture information from women across the country, an online survey was selected as a convenient method to collect data.

### Sample and setting

Women who had received all their maternity care to six weeks postnatal in any Australian state or territory from 1 January 2020 to 30 June 2023 were eligible to participate in the survey. If a woman had given birth to more than one baby in that time, she was invited to complete the survey about the maternity care received for her most recent baby. All Australian maternity care services, including any model of private or public maternity care within the Australian health care system, provided the setting for the study. Women were asked to report their model of care according to the Maternity Care Classification System [4] (Table 1).

### Survey

The survey consisted of demographic information, clinical information, and the Australian Maternity Care Values Assessment Tool [12]. The tool was developed for the purposes of this study so that women’s maternity care experiences could be assessed according to the principles and values of the Strategy. The survey content was designed by three expert health professionals (PM, LS & AH) using available evidence. A group of ten consumers, which included equal representation from the named priority populations in the Strategy, pre-tested and refined the questions. This ascertained consumer acceptability and understanding of the survey and enhanced content validity. The survey was additionally translated into Chinese (Simplified) and Arabic, as these are the two most widely spoken languages in Australia after English [15]. Prior to distribution, the reliability of the English version of the survey was verified via a test-retest using Cronbach’s Alpha [12]. This was 0.89, considered high [16].

For reference, women were provided with an electronic link to the Strategy on the opening page of the survey. The survey consisted of 62 questions, of which six were open-ended, allowing participants to provide in-depth answers if desired. Of the open-ended questions, four were about the values of the Strategy, ‘Do you have any further comments you would like to make about the value of safety/respect/choice/access,’(respectively) and one question was about the Strategy itself, ‘Do you have any overall comments you would like to make about the national maternity care Strategy?’ There was one further question, ‘Are you able to provide more detail on why and at what point you felt unsafe,’ which was asked (using skip logic) only if the participant indicated that they had felt unsafe one or more times throughout their maternity care. From these questions, qualitative data were collected.

### Data collection and analysis

The survey was advertised intermittently on Facebook between February and June 2023 and engaged advertising metrics to target women with infants and young children who resided in all Australian states and territories. The Qualtrics platform was used to host the survey, and access was gained via a hyperlink or QR code contained in the Facebook advertisement. It is likely that snowballing of the sample occurred due to the ease of sharing the advertisement via social media.

The qualitative analysis design was based on the seven stages of Nicmanis’ reflexive content analysis, where reflexivity is central to each stage of the analysis process and assists to strengthen the development of the research findings [17]. The seven stages are refining the research question, data collection and familiarisation, coding, revising codes, developing analysis structure, reporting the analysis structure, and interpreting the findings [17].

Survey responses were included in the analysis if the eligibility criteria were met, consent was provided, and more than 50% of the survey was completed. To maintain participant confidentiality, any identifying information collected in the free text boxes was removed prior to analysis. Qualitative data were then imported into NVivo software version 14 [18] and a reflexive content analysis [17] undertaken by coding, revising the codes, and combining these to create sub-categories, using the four values in the Strategy as overarching categories. Coding and sub-categorisation development were initially undertaken by one author (PM), then refined and developed in conjunction with other members of the research team (LS, AH). Reflexivity was undertaken at all stages of the process. All three authors have been educated in midwifery, and the first author is a practising midwife. All three had to bracket their experiences, emotions and feelings to engage objectively with the data. A reflexive journal was used during the data analysis by the first author to ensure neutrality so that personal predispositions did not compromise objectivity. The research team also held regular, structured reflexive discussions to spark collaborative reflexivity [19] and to examine how the coding process was evolving.

### Ethics

Ethical approval was gained on 21 September 2022 from the Deakin University Human Research Ethics Committee, approval number 2022-217. Electronic informed consent was obtained from participants prior to commencing the survey and participants only accessed the survey if they consented to proceed. Participation was not incentivised, was entirely voluntary and anonymous. Women could withdraw at any time while completing the survey however once their survey responses were submitted, withdrawal was not possible due to the anonymity of the responses.

## Results

The survey yielded 1750 usable responses, and of these, 1667 participants provided one or more qualitative responses included in this analysis. All Australian states and territories were represented in the survey, and participation was proportionate to each jurisdiction’s population, with a slight over-representation from the state of Victoria (Table 2). The mean participant age was 33.8 years, and over half (55.1%) were first-time mothers. Home location was consistent with national data and indicated that 81.4% of women resided in metropolitan or regional areas, 16.4% in rural areas, and 2.2% lived remotely [20]. Private obstetric specialist care was the most common model of maternity care accessed (26.2%), followed by public hospital maternity care (22.7%) and MGP (15.7%). Combined household incomes in the sample were higher than in the general population [21] as were the proportion of women who were tertiary educated [22].

**Table 2:** Characteristics of study sample (*N* = 1667).

Jurisdiction where maternity care was received	Frequency	
*n*	%
Australian Capital Territory	91	5.5
New South Wales	410	24.6
Northern Territory	16	1.0
Queensland	254	15.2
South Australia	166	10.0
Tasmania	38	2.3
Victoria	547	32.9
Western Australia	145	8.7
**Age in years**		
25 or younger	41	2.5
26-33	745	44.7
34-40	784	47.0
41 or older	97	5.8
**Parity**		
Primiparous	919	55.1
Multiparous	748	44.9
**Aboriginality**		
Identifies as Aboriginal and/or Torres Strait Islander	20	1.2
Does not identify as Aboriginal and/or Torres Strait Islander	1647	98.8
**Culturally and linguistically diverse identification**		
Identifies as culturally and linguistically diverse	117	7.0
Does not identify as culturally and linguistically diverse	1550	93.0
Rurality*		
Metropolitan and regional	1355	81.4
Rural	273	16.4
Remote	37	2.2
**Educational attainment**		
Year 12 or less	80	4.8
Certificate or diploma	210	12.6
Bachelor degree and/or graduate diploma	888	53.2
Master’s or doctoral degree I prefer not to say	484 5	29.0 0.3
**Combined yearly household income (AUD)**		
$75 000 or less	115	6.8
$75 001 - $125 000	384	23.0
$125 001 - $200 000	685	41.1
$200 001 or more	422	25.3
I prefer not to say	61	3.7
Model of maternity care*		
Private obstetrician care	437	26.2
Public hospital care	379	22.7
Midwifery group practice	261	15.7
Public hospital high risk care	167	10.0
Shared care	166	9.9
Private midwifery care	82	4.9
Team midwifery care	55	3.3
General practitioner obstetrician care	44	2.6
Private obstetrician and private midwife joint care	41	2.5
Combined care	31	1.9
Remote area care	3	0.2

* Missing values: rurality (*n*=2), model of maternity care (*n*=1).

**Fig 1:**
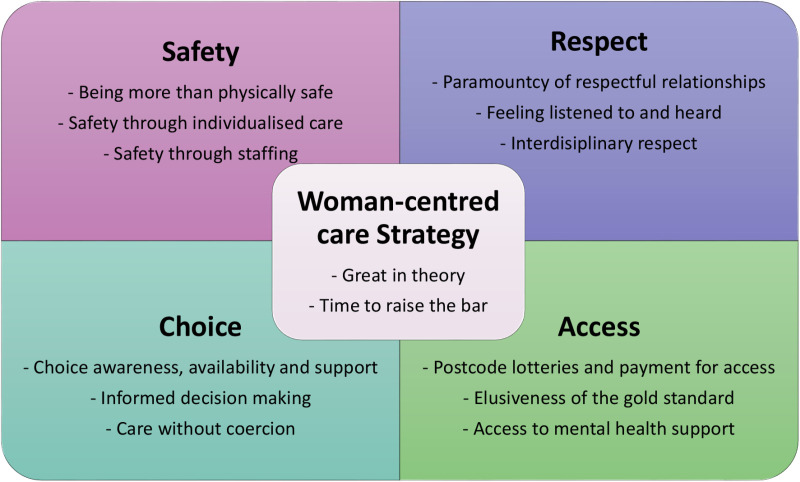
Summary of main findings.

There were 3663 comments made in the six open-text boxes in the survey. Data were cleaned prior to analysis by removing responses that did not provide further information, such as ‘N/A,’ ‘nil,’ or ‘no.’ There were 101 such comments, meaning 3562 comments were included in the analysis. In total, there were 795 comments from women detailing what aspect of their maternity care made them feel unsafe, 605 general comments about the value of safety, 466 about respect, 637 about choice, 476 about access, and 583 overall comments about the Strategy itself. Although eight surveys were completed in the Simplified Chinese or Arabic versions, none of these included open-text comments, so the included data were recorded exclusively in English. Whilst each of the four values framed the analysis, there were three sub-themes developed from the women’s responses. A summary of the main findings can be seen in Fig 1, where the centrality of woman-centredness to the Strategy is shown, encompassed by the four equally weighted values.

### Safety

The value of safety in the Strategy upholds that women should have individualised and appropriate care based on high-quality evidence from a responsive, competent (including culturally competent) workforce that is well-resourced [2]. Three sub-themes were derived from data from the two survey questions relating to safety: ‘*being more than physically safe*’, ‘*safety through individualised care*’, and ‘*safety through staffing’.*

#### Being more than physically safe.

The free-text survey responses demonstrated that women prioritised safety for themselves and their infants across their maternity care episode; however, emphasised the need for a holistic approach to safety, and the need to feel more than physically safe. In other words, women valued care that made them feel emotionally and psychologically safe just as much (and sometimes more than) care that provided physical safety. Women indicated that physical safety was often prioritised at the expense of other non-physical safety measures, contributing to an overall sense of feeling unsafe. Further, the provision of maternity care that was not emotionally or psychologically safe was often reported as leading to ongoing trauma postnatally, sometimes resulting in the need for women to seek mental health support. While it was acknowledged that Australia is generally considered a safe place to give birth, this was only with respect to physical safety, as stated by these participants:

*Physical safety when giving birth in Australia is almost a given; our outcomes are similar no matter the place of birth. When talking about emotional, psychological, and feeling a sense of safety, that is very hard to find inside a hospital. (ID 928)*

*I think there needs to be a real effort to recognise that quality maternity care does not mean only providing safe physical care for women and their babies. Pregnancy, birth, and postpartum are often emotionally difficult and overwhelming, even in the best of circumstances. Expecting women to soldier through so much of these challenges alone is unacceptable and retrograde in 2023. Australia has one of the best healthcare systems in the world. Time to raise the bar on maternity services so that they also fit this description. (ID 217)*

Further, the concept of safety was viewed as an individual measure that should be defined by the woman herself:

*To me, safety refers not just to clinical knowledge and competency but also to the care that is provided with respect and with the goal of empowering women. Safety is defined by the woman receiving the care, and informed consent (without coercion or judgement) is an absolute necessity… to allow women to define what is safe for them and the level of risk they are comfortable with. (ID 1671)*

#### Safety through individualised care.

The second sub-theme speaks to the importance of individualised maternity care and information provision to enhance women’s sense of safety, and how safety becomes compromised if this is lacking. Women who received care from multiple providers were more vulnerable to feeling that their safety was jeopardised, especially if conflicting information was provided by different health professionals, as described below:

*I often felt judged throughout antenatal appointments and did not ask questions or engage in my care because I felt brushed off… like the midwife had no time for me. I did not necessarily believe the midwife’s information because most people gave differing information, and this made me feel extremely unsafe and uncomfortable, and instead of being cared for during my pregnancy made me feel like a box for the staff to tick. (ID 717).*

*I never saw the same midwife or doctor more than once. Each antenatal visit was someone new and every medical professional had a different opinion and spoke about different risks or things to worry about that when they were compounded made me very anxious. I didn’t have just one person to call on or trust. (ID 250).*

Continuity of care with a known maternity care provider mitigated the risk of feeling unsafe, as the following women illustrate:

*I felt incredibly safe with my obgyn [obstetrician-gynaecologist] and was able to have many discussions with her around the safety of my pregnancy and birth. I felt like my wishes were taken into account, and then also considered with safety in mind. (ID 889)*

*Having a known midwife to myself and my family helped to make myself and partner feel safe, it was someone we trusted to inform, educate, and assist in making the right decisions whilst allowing me to listen and follow my own intuition. Was amazing care. (ID 790)*

#### Safety through staffing.

In the third sub-theme, participants described workforce shortages that were prevalent across the Australian maternity care system, particularly during the inpatient stay for intrapartum and immediate postnatal care. Staffing shortages in health care facilities were noticed by women in both public and private facilities, and this compromised their sense of safety:

*Not having enough staff made it impossible to feel safe. (ID 339)*

*After the baby was born there was an extreme lack of assistance from midwives in the hospital. Midwives were only seen once a shift, and when asking for assistance nil was provided. I felt unsafe with the advice or lack of advice/assistance. (ID 505)*

*I was pushed into having an induction as there were concerns I had IUGR [intrauterine growth restriction]. I gave birth to a perfectly healthy baby. What felt unsafe was that I was told the induction was urgent and had to be done that day. I asked what was the latest we could leave it to see if I would go into spontaneous labour and was told we cannot leave it later. I was then told (due to staffing) they couldn’t book me in for an induction for 4 days. (ID 166)*

### Respect

The value of respect in the Strategy describes holistic care, where women are always treated with dignity and respect, and where the woman’s emotional, psychosocial, spiritual, and cultural needs are met by maternity care providers. This value also requires maternity care to be underpinned by respectful communication and collaboration among health professionals [2]. Three sub-themes were observed within the value of respect: the ‘*paramountcy of respectful relationships’, ‘feeling listened to and heard’,* and *‘interdisciplinary respect’.*

#### Paramountcy of respectful relationships.

Respectful relationships with maternity care providers were highly valued by survey participants, and where this occurred, women felt empowered to make autonomous and informed decisions for themselves and their babies. Women described the need to have their decisions respected by their maternity care providers and the importance of being guided through the decision-making process in a way that valued the woman’s opinions and wishes. When this did not occur, women lacked trust in their care providers, and the therapeutic relationship became strained:

*I am an intelligent, educated woman who just happened to be pregnant and giving birth. There seemed to be an attitude amongst caregivers that this diminished my ability to understand the risks of a certain situation or to make a rational decision. It feels very disrespectful of my status as a rational and capable adult, and anytime I spoke up, I was treated like a naughty child in a classroom. (ID 733)*

*With only one exception, I was spoken AT by doctors who didn’t take the time to find out my… background… or my appetite for risk. I could quote relevant statistics re my ‘high-risk’ pregnancy (gestational diabetes) better than they could, so I had the capacity to make my own decisions. Apart from one obstetrician, they didn’t seem to get that not all pregnant people want a ‘healthy baby above everything else.’ I was willing to tolerate a miniscule increase in risk to my child (late term stillbirth) to have a dramatically better chance of having my desired birth (vaginal birth with no intervention). I was borderline yelled at by an obviously contemptuous registrar for refusing an induction. (ID 2)*

#### Feeling listened to and heard.

Throughout the survey, the importance of being listened to and heard by their maternity care provider was identified as a means of generating respect within the therapeutic relationship. It also served to ensure the woman felt in control of her maternity care, increased satisfaction with the care provided, and even served as a deterrent to negative emotions:

*During pregnancy and birth, I was listened to at all stages, they answered all of my questions and asked for consent. It allowed me to make decisions and walk away from what could have been a traumatic birth, feeling comfortable and empowered. (ID 135)*

*I felt that my midwives and doctors always respected my decisions about my body even when I made requests that went against standard practice. This helped me to feel confident, calm, and psychologically safe. (ID 1286)*

Conversely, not feeling listened to or heard by maternity care providers had a negative effect on the perception of care received, as stated below:

*Providers need to LISTEN to women and not treat them like they don’t know what they’re experiencing and just placate them but go with their own agenda anyway. (ID 323)*

*I didn’t feel reassured by the midwives when I expressed concern or a preference for my birth. I didn’t feel heard, and being a first-time mum, I felt lost, and didn’t know what guidance I needed, what to expect in a public hospital birth, what… process to follow. (ID 996)*

In addition, perceptions of being unheard could remain with women long after the postnatal period was complete and contributed to symptoms of birth trauma, as described by this woman:

*Feeling unsafe and unheard during my pregnancy, birth, and postnatal period has lingered with me and I often find myself thinking about it in the middle of the night and replaying conversations and situations with my OB [obstetrician] and midwives and feeling angry and upset. (ID 930)*

#### Interdisciplinary respect.

The interplay of relationships between different maternity care providers was observed by women participating in the survey, who noticed particularly where their relationship between providers was strained due to differing opinions of how care should be provided. This compromised women’s perceptions of respectful care, and they felt caught in the middle of the negative interdisciplinary interactions:

*My obgyn [obstetrician-gynaecologist] and a midwife got in a fight in front of me. He was upset that she had not removed my catheter or helped me shower the day after my c-section [caesarean birth]. When he requested materials to remove my catheter, she got them and then left the room without assisting the doctor. They both complained about the other person separately in front of me. It was very awkward. (ID 363)*

Further, the differing philosophy of care provision between medical practitioners and midwives was noticed by women and was described as confusing and unhelpful:

*While my MGP midwife was excellent, there can sometimes be a bit of shade thrown between midwives and obstetricians (in both directions). The us versus them vibe is extremely unhelpful and makes all the material from both sides read like propaganda. (ID 1724)*

*There is still ongoing disagreement between doctor and midwife approaches to maternity care, I very much dislike passing on a comment from one member and having it disparaged by the other member. (ID 429)*

### Choice

The value of choice states that women should have ready access to information about locally available maternity services, that upholds the principle that women should be supported to make informed decisions about their care, and that women’s choice should be sought and upheld by maternity care providers [2]. From the value of choice, three sub-themes were identified: ‘*choice awareness, availability and support’, ‘informed decision making’,* and *‘coerced care’.*

#### Choice awareness, availability, and support.

Universally, women described a lack of awareness about the different maternity models of care locally available to them. They further outlined the requirement to choose a model of care in early pregnancy with little information provided about how the models worked and how this decision would later impact care provision across the maternity care episode. A lack of awareness meant that women often missed out on their model of choice, particularly public MGP models, as by the time they became aware of them, the models were already fully booked. Women also described the gatekeeping function of the general practitioner (GP) in guiding their decision regarding the model of care choice, which was often simplified into a decision between the public and private health systems. The following are examples of this situation:

*I was most of the way through my pregnancy before even understanding the differences in models of care. Women are expected to lock in decisions very early in pregnancy without understanding the implications of those decisions on their care. (ID 1251)*

*The survey lists 11 forms of maternity care, but I didn’t get this list and wasn’t asked which one I would prefer… I was asked if I was going public or private and if I would choose shared care with my GP. That’s it. (ID 135)*

*It would be nice if medical professionals outlined ALL the available choices, not just those they personally agree with. My GP neglected to mention that home birth or private midwifery care were options, so it wasn’t until halfway through my pregnancy I had done all my research and changed to this option. I felt like I had to present all my reasoning to the GP for her to write a referral letter though, and she told me she hopes I don’t come back to her with birth trauma when I don’t get the magical water birth I want. Zero evidenced based advice there. (ID 344)*

Women also discussed the need to have their care choices supported by their maternity care providers as it enabled them to feel empowered, respected, and in control of their decision making and care. Conversely, a lack of support for a woman’s decision had the opposite effect. The following two statements provide examples of how positive and negative support impacted choice:

*I was pleasantly surprised that despite my birth not going at all to plan (unexpected home birth to a frank breech baby, no medical assistance present during) - paramedics, ED [emergency department] staff, and maternity staff all took the time to read my birth preferences and respected my choices. That gave me back a small amount of control in a very uncontrollable situation and it was much appreciated. (ID 808)*

*The first obstetrician who cared for me through my pregnancy was initially supportive of my wishes for a VBAC [vaginal birth after caesarean] as there were no medical reasons why I needed a repeat caesarean. Late in the pregnancy, he started to voice his opinions strongly about his preference for caesarean and imposed a lot of ‘conditions’ on my being ‘allowed’ to give birth vaginally. He became condescending and dismissive towards me when I asked for evidence-based information to support his preferences. I felt unsupported and abandoned. (ID 134)*

#### Informed decision making.

Survey respondents stressed the importance of being given appropriate information to inform maternity care decision-making processes; however, many felt that this was often not provided by clinicians. This resulted in women consenting to procedures for themselves or their baby, which they later considered as provided without being truly informed. They also described being forced to ‘choose’ what their maternity care provider wanted for them rather than making the decision freely for themselves, and how this impacted their emotional well-being and sense of autonomy:

*I never felt like I was provided with enough information... I feel like they purposely keep you a little bit in the dark so you can’t stand up for yourself and advocate for yourself, so if it comes down to it, they get to make that decision for what suits them best, rather than giving you all you need to decide for yourself. (ID 1676).*

*I felt like I was nominally given choice (e.g., sign here… for your emergency c section), but these were not real choices, as I wasn’t the best-informed person to know whether these procedures were truly necessary. I felt extremely disempowered and in many ways, the fact that these things were my ‘choice’ (but not really), made the whole thing emotionally much worse. (ID 1703).*

*I was not educated at all about relevant medical options when pregnant, nor ever asked or discussed different options for a birth plan. Procedures were done and medications administered to me… that I did not consent to or have explained to me beforehand. I felt powerless because of it. (ID 930).*

Contrarily, women described the positive impact of informed decision-making, even when care deviated from expected outcomes:

*I felt very informed about all my choices. Things didn’t go as I had hoped, but I feel absolutely fine about it because I was informed, felt in control, and not pressured. (ID 1624)*

#### Coerced care.

Experiences of coercion in maternity care were commonly reported in the survey. Women described how feeling coerced by their maternity care providers negatively impacted their autonomy and preferences and led to tension:

*I did NOT feel safe when in the presence of obstetricians, who pulled the ‘dead baby card’ without any evidence on two occasions. They clearly were… so convinced my baby was in danger that they made me sign an acknowledgement that I was refusing medical treatment. It was clear to me that they were just trying to coerce me into accepting an intervention (induction of labour) that, a) was risky, and b) was unnecessary. (ID 1093).*

*A jaded midwife ignored my requests for assistance, help with positioning, and general support during labour. I was left in unnecessary pain, and she ignored my birth plan wishes and coerced me into procedures I clearly didn’t want, all to make her shift at work easier… I felt dismissed, powerless, and still suffer physically 18 months on with still no answers as to what is causing my ongoing pain. (ID 268)*

### Access

The value of access promotes improved access to maternity care in a location of choice from conception until 12 months postnatal. Improved access to continuity of care and mental health support are also found within this value [2]. Sub-themes generated from the access value were ‘*postcode lotteries and paying for access,’ ‘elusiveness of the gold standard*,’ and ‘*access to mental health support’.*

#### Postcode lotteries and payment for access.

Issues of access were commonly referred to in the survey responses, where women frequently described difficulty in accessing the care they wanted and needed, with a ‘*postcode lottery*’ seen as a possible explanation. Paying for care was seen as a solution to mitigate access problems, although, as one respondent suggested, *‘what you can afford determines your level of care and options available to you’* (ID 1246). Accessing care by paying for it was also acknowledged as a privilege and one that many could not afford. The following quotes illustrate these points:

*Currently, it feels like a postcode lottery to access appropriate care. I got lucky and live in a reasonably well-serviced area. Friends and family that live only 15 minutes away had different experiences of care due to the postcode lottery. (ID 267)*

*I am very privileged to be located in (capital city), to be able to afford private health insurance and out-of-pocket costs for private medical and allied health services such as obstetrician/lactation consultant/postnatal physiotherapist, to speak English, and to have high health literacy, and access to informal medical advice via medical friends and medical social media groups. If any of these factors were flipped the other way, they would be barriers to accessing care. (ID 1716)*

*Private midwifery care is a good standard, and it costs a lot of money, however, it should be the minimum standard. My care was exceptional and helped me heal from a lot of previous traumas. Many women in Australia cannot afford this kind of care and therefore have no access to it. The Medicare and private health rebates are so low or non-existent it makes this type of care only accessible to the wealthy. (ID 1553)*

#### Elusiveness of the gold standard.

Access to continuity of care with a care provider of choice, including midwifery continuity of care, is one of the principles in the Strategy. Although this was universally referred to as the ‘*gold standard*’ of maternity care and highly desired by women in the survey, women also described their difficulty in accessing midwifery continuity models. Private midwifery care was considered more accessible but was also seen as expensive and for some, cost prohibitive. Public MGPs were considered almost impossible to access due to the high demand exceeding availability. Women also described their disappointment at being ineligible to access MGP if deemed to be ‘at risk’, as some MGP models exclusively cater to low-risk women. The following quotes illustrate the situation:

*Caseload midwife care is the gold standard. It’s an embarrassment to our healthcare system that all women can’t access this. It shows how undervalued women’s health is. (ID 88)*

*Accessing continuity of care in my area through the public hospital is almost impossible - not enough midwives in the program and too many women wanting access. We chose private midwifery for a number of reasons, but this was a very expensive option and definitely was a big investment, one that many simply cannot afford. (ID 1739)*

*Many women, like myself, who desire & apply for midwifery continuity, are unable to access it. I did not get in last pregnancy. Now pregnant again, it is not even an option for me to apply, because I am ‘high-risk’. It should be a priority for the government to make both public & private midwifery continuity models widely available for all women of all risk categories who wish to receive an evidence-based best model of care. (ID 320)*

#### Access to mental health support.

A further principle of the Strategy is that women should have access to mental health information, support, and treatment from conception until 12 months after the birth. The findings from the survey demonstrated that perinatal mental health support was difficult to access, as described by these women:

*I called EVERY service imaginable for help and some places didn’t even answer the phone. A mental health nurse said that sometimes you just need to ‘give up trying.’ I even went to a mental health ward in (tertiary hospital) to get some help and they just sent me home and said to think happy thoughts. (ID 1210)*

*Mental health care was the most difficult to access. The very long wait lists to access a GP for a mental health plan and then a very very long waitlist to see a psychologist was… difficult. By the time I saw the psychologist, I was… unwell to the point my husband had to take time off work to look after my baby and I. (ID 147)*

### Comments about the strategy

From the open-ended question inviting women to provide overall commentary about the Strategy, two main themes were identified: ‘*great in theory’,* and ‘*time to raise the bar’*.

#### Great in theory.

The appropriateness of the Strategy for guiding Australian maternity care was generally acknowledged by respondents, but it was universally agreed that the Strategy had not been well-implemented or translated into practice. The following comments were typical examples relating to this theme:

*Great in theory, but health systems and health professionals don’t follow it in the real world. (ID 1744)*

*It seems a lot of some very nice words… women are being let down, especially in relation to respect and choice. The Strategy doesn’t line up with our birth stats. (ID 927)*

#### Time to raise the bar.

The second theme arising from the general commentary calls on Australian maternity services to elevate the Strategy from a strategic document to an operational policy where women experience transformational maternity care as intended. The following comments illustrate this desire for change:

*My experience in the maternity system makes me feel that it is almost luck to receive care that is in line with the maternity care strategy… The Strategy is not widely known by pregnant people and if it were more clearly stated I feel women would be able to know better when they are receiving care that is not in line with it. (ID 1676)*

*Mums matter! Mental health matters! We are not just incubators. A healthy baby is essential and always the outcome to be aiming for, but it cannot be the only goal of a birth experience. We need to start thinking about how to make this work for the people giving birth so that we are not coming home traumatised and afraid. (ID 733)*

*It desperately needs to be more transparent, accessible and visible for ALL consumers of maternity care. If we don’t know about what’s available, nothing will change! (ID 712)*

## Discussion

This paper presents the qualitative findings from a national survey of geographically representative women who had received maternity care in Australia since the release of the national maternity Strategy. The findings demonstrate that, despite its release over five years ago, women are not always receiving care according to the expressed values and principles in the Strategy. This is regardless of a desire by women to receive care as the Strategy articulates. It is also despite a commitment from the state, territory, and Commonwealth governments to progress the Strategy. While previous research suggests that implementation has been slow [8], the findings of this study confirmed this view, and indicate that the Strategy is not influencing the provision of quality maternity services at the point of care.

Women in Australia value safety in maternity care [7], and while the Strategy describes Australia as ‘a safe country in which to have a baby’ [2 p.4], the safety and quality measures routinely used to report on safety [1] do so entirely based on measures of physical safety. They do not capture the high rates of women feeling emotionally or psychologically unsafe [12,23] or consider reports of birth trauma experienced by women who access Australian maternity services [24]. While high rates of birth trauma and obstetric violence persist [23,25], Australia cannot be considered a safe country in which to have a baby (taking safety in the holistic sense). The women participating in this survey expressed a desire to move to a more holistic and encompassing definition of safety rather than focussing on physical safety alone, with emotional and psychological safety prioritised throughout the maternity care episode. With a move towards the use of patient-reported outcome measures (PROMs) and patient-reported experience measures (PREMs) [26], a re-examination of how safety is framed in Australian maternity services needs to be considered. Internationally the use of PROMs and PREMs has been found to provide deeper insights into how women perceive the quality of their maternity care [27], and routine use has demonstrated an increase in woman-centredness [28]. Adaptation of a validated and uniform set of national PROMs and PREMs therefore has the potential to provide maternity care clinicians and policy makers alike with measurable metrics to facilitate woman-centredness and increase women’s sense of safety system-wide. Linking these to additional funding for states and territories who perform well on these measures has the potential to raise the watermark of woman-centredness even higher.

The maternity care workforce is facing critical shortages in Australia, particularly in the midwifery profession, where, despite a growing Australian population, there are fewer midwives on the national register than there were five years ago [29]. While consideration is being given nationally to how to address these workforce shortages [29–31], this research indicates that while shortages continue, they will contribute to compromising the sense of safety in Australian maternity services.

This study highlights the paramountcy of relationship-based maternity care, and its importance in cultivating respect within therapeutic relationships, which empowers women to feel in control of their maternity care choices and outcomes. It builds on the work of previous studies in Australia, and internationally [32,33] that also demonstrate the value women placed on being seen and heard, empowered to make autonomous decisions, and to have those decisions respected by their maternity care providers [7,34–36]. The findings of this study also reinforce that women perceive this is best achieved through continuity of care models, particularly those offering continuity with a known midwife, which have well-demonstrated benefits [37–40] and are highly valued by women [6,26]. Yet despite a large body of evidence demonstrating as such [24–27], Australian public maternity services consistently fail to meet the demand for continuity of care models, forcing women who have the means into private models to receive continuity, and those who do not into models without continuity. This demonstrates inequity within Australian maternity services and contravenes the principle in the Strategy of having ‘access to continuity of care with the care provider(s) of… choice’ [2 p.16].

The importance placed on having the agency to make informed decisions, free from coercion, across maternity care has been highlighted by this research, building on the findings of previous Australian studies [7,12,34,41]. This study shows that despite an articulated desire, coercion, and lack of information to inform decision-making persists, breaching the value of choice and its associated principles in the Strategy. An additional finding of this research is that decisions made in early pregnancy, when women are least aware of their care options, can impact the entire span of the maternity care episode, particularly in relation to the model of care choice. Women need to have maternity care information readily available, easily accessible, and widely advertised, to enable early decision-making, particularly around models of care. It is suggested in the Strategy that the Australian government website ‘Pregnancy Birth and Baby [42]’ is ideally placed to host model of care information [2], but at the time of writing, a clear description of Australian maternity care models and how they are accessed has not yet been provided. Until maternity care information is universally accessible, women and particularly first-time mothers will continue to experience inequity in model of care access, inhibiting choice.

The findings of this research demonstrate that maternity service access is not equitable, despite the Strategy stating that women should have access to care in their location of choice with their provider of choice [2]. Further, it shows that the Australian health care system frequently fails women who require access to mental health care in the perinatal period, despite known rates of birth trauma [11,24]. It highlights that a divide exists between those who can afford to pay for care and those who cannot. Australia’s overarching public health scheme, Medicare, is based on the principle of universal access, which enables all citizens and residents to access a wide range of health services and medicines at little or no cost [43]. Despite this, maternity care access issues are widely described in Australian literature, especially among women from Australia’s priority populations [7,44–46]. Again, the surveyed women found that the principle of universal access to maternity services through the public system is not currently meeting the needs of many women in Australia. Deficits in accessing mental health support were perceived as particularly problematic and occur despite the investment that successive Australian governments have given to perinatal mental health initiatives [47–49].

Finally, participants in this study articulated a desire to know more about the national maternity care Strategy and to advocate for and receive care that aligns with its values and principles. Awareness, therefore, needs to be raised among consumers of Australian maternity services about the existence of the Strategy so that there is an expectation that care will be received accordingly.

## Strengths and limitations

A strength of this study is that it is the first of its kind to directly compare the values and principles of the Strategy with the maternity care experiences of women. The qualitative data gives voice to the consumers of Australian maternity services who are the end-users of the Strategy. The findings provide a first-hand insight into the ways in which the Strategy is working while highlighting areas for improvement. The large number of participants and qualitative data collected strengthen confidence in the study findings and provide rigour to the interpretation.

This study has several limitations. As the Strategy was released just prior to the COVID-19 pandemic, the results are likely impacted by the challenges to resource allocation and staffing that were universally faced by Australian maternity services at that time [50]. Further, while survey participants were geographically representative, they were more educated, had higher household incomes, and were less representative of women from diverse cultural backgrounds than the general Australian population, so the results are potentially not transferable. Additionally, women self-selected to participate in the survey so possibly held strong views on the quality of maternity care received, which may have biased the results. Even if the woman declared before entering the survey that they became pregnant after January 1 2020, there was also potential that women giving birth before this time may have accessed the survey as it relied on participants to honestly declare they met the selection criteria.

## Conclusion

Australia’s national maternity Strategy, *Woman-centred care: Strategic directions for Australian maternity services* has been the guiding document for the provision of woman-centred maternity care nationwide since its release in late 2019. This study has given voice to women who have received maternity care in Australia following its release and who have described ways in which the Strategy aligns and misaligns with their experiences of care. It shows that despite a shared responsibility by Australia’s states and territories to implement the Strategy, a greater commitment is required nationally if the Strategy is to move from an unrealised policy to universally transformative care across Australian maternity services.

## Terminology

The terminology in this research is gendered. We acknowledge that not every maternity care consumer in Australia identifies as a woman, and we respect each individual’s preferred terminology. The terms ‘woman’ and ‘women’ have been used in this paper for consistency as this is the language used in the national maternity Strategy.
